# Changes in Urinary 8-Hydroxydeoxyguanosine Levels During Heptathlon Race in Professional Female Athletes

**DOI:** 10.2478/hukin-2014-0038

**Published:** 2014-07-08

**Authors:** Badiea Ali Abdel Samia, Gehan Ahmed Youssef

**Affiliations:** 1 Faculty of physical education for girls, Helwan University, Egypt.; 2 Faculty of medicine, Al Azhar University, Cairo, Egypt.

**Keywords:** heptathlon, oxidative DNA damage, 8-hydroxydeoxyguanosine (8-OHdG), malondialdehyde (MDA), oxidative stress

## Abstract

Acute strenuous exercise can induce a state of oxidative stress affecting the involved muscles. Heptathlon is a multi-event exercise of two days duration and can be considered an acute, intensive endurance exercise. The purpose of this study is to compare the oxidative stress response to heptathlon events day by day and to determine the impact of this type of exercise on oxidative stress biomarkers.

The study subjects included eight heptathlon athletes who participated in the National First Class Republic competition in Egypt (October 19–21, 2011). Blood samples were collected at rest after exercise for two successive days and analyzed for malondialdehyde (MDA). Morning urine samples were collected one hour after exercise for each day and were analyzed for 8-hydroxydeoxyguanosine (8-OHdG). Results revealed a significant increase (p<0.05) in both plasma MDA and urine 8-OHdG levels after exercise regardless of the day. We concluded that exercise generates higher MDA levels compared to DNA strand breaks and oxidative DNA damage in athletes with antioxidant supplementation.

## Introduction

It has been documented that acute bouts of aerobic and anaerobic exercise can induce a state of oxidative stress as indicated by an increase in oxidized molecules in various tissues and body fluids. The extent of oxidation is dependent on the exercise mode, intensity, and duration and is specifically related to the degree of oxidant production. Findings of increased oxidative stress have been reported for both healthy and diseased subjects following single bouts of exercise. While acute exercise has the ability to induce oxidative stress, the same exercise stimulus appears necessary for upregulation of endogenous antioxidant defenses (Bloomer, 2008).

Physical exercise causes a temporary muscle ischemia, leading to cellular hypoxia. At the end of exercise, there is an increased oxygen supply during recovery as a result of reperfusion. This sudden influx of oxygen causes calcium overload in cells, leading to an influx of inflammatory cells into reperfused tissue. This leads to the generation of reactive oxygen radicals causing oxidative damage to DNA, proteins, and lipids, further leading to cellular damage (Martarelli and Pompei, 2009; Powers and Jackson, 2008). Exercise-induced oxidative damage in tissue can damage cellular membranes, induce cellular swelling, decrease cell membrane fluidity, prevent maintenance of ionic gradients, and lead to tissue inflammation, DNA damage, and protein changes. All of this can result in fatigue, delayed-onset muscle soreness, and increased injury recovery times (Alessio et al., 2000; Davies et al., 1982; Halliwell and Cross, 1994).

Prolonged heavy exercise may cause a transient reduction in tissue vitamin E content and a change in glutathione redox status in various body tissue leading to exercise-induced oxidative stress and tissue damage (Banerjee et al., 2003). Both aerobic and anaerobic exercise protocols increased oxidative stress to a similar extent (Bloomer and Smith, 2009). A single bout of exercise, depending on intensity and duration, can cause an increase in antioxidant enzyme activity and decrease levels of thiols and antioxidant vitamins with the net result of oxidative damage. Increased levels of free radicals and oxidative damage are initiators of a specific adaptive response, such as the activation of antioxidant enzymes, thiols, and enhanced oxidative damage repair (Radak et al., 2001).

There are many previous studies investigating plasma peroxidation markers as indicators of tissue damage, but recently other studies have revealed such markers as falsely positive due to the loss of the facultative permeability of cell membranes. Therefore, it is recommended that future investigations employ sufficiently stringent exercise protocols and utilize a wide array of oxidative stress biomarkers (Fisher-Wellman et al., 2009).

Moreover, it seems that none of the studies investigated multi-event sport disciplines. Heptathlon is one of these sports, requiring intermittent spurts of effort and recurrent heavy exercise within a couple of days.

The aim of this study was to investigate the effect of heptathlon competition on the urinary excretion of 8-hydroxy-deoxyguanosine (8OHdG) from elite athletes as compared to the traditional oxidative stress marker, malondialdehyde (MDA).

## Material and Methods

### Subjects

Eight female heptathlon athletes participated in the National First Class Republic competition in Egypt (October 19–21, 2011) and constituted the subjects of this study. None of the participants smoked, took medications, or received supplements. There was no clinical or analytical evidence for any disease among the participants. The effects of four events performed in the first day were studied. These consisted of the 100m run, high jump, shot put, and 200m run. On the second day, the athletes participated in the long jump, javelin throw, and 800m run. All events had a 35min inter-period. The competition started at 3p.m. and ended at 6 p.m. on the first day, while on the second day, it started at 8a.m. and ended at 10a.m..

### Blood analyses

Blood samples were collected before and immediately after exercise during the two days. They were collected in heparinized tubes and centrifugated at 1000–1500g for 10 minutes. Plasma was separated and frozen at −20°C until analysis was performed. MDA levels were measured using a lipid peroxidation assay kit (Calbiochem, San Diego, CA) (Esterbauer and Cheeseman, 1990).

### Urine analyses

A morning urine sample (∼50ml) was collected one hour after exercise for the two successive days. Urine samples were analyzed for 8-hydroxydeoxyguanosine (8-OHdG) using a monoclonal antibody-based ELISA kit (Genox, Baltimore, MD) (Shigenga et al., 1989).

### Statistical analysis

A Computer program (SPSS version 15) was used to analyze the obtained results. Student’s Mean, standard deviation of athletes, and the Wilcoxon Sign Ranks Test were used to examine the differences between baseline and post-exercise measurements for each day. The level of significance was set at p≤0.05.

## Results

Anthropometric variables of study participants (age, body mass, body height, and training experience) are presented in Table 1. All values of skewness lie between −3 and +3, which indicates the equivalence of the subjects.

Plasma MDA and urinary 8OHdG levels were elevated after exercise when compared to those obtained before exercise, implying a significant increase of both markers of oxidative stress of competition during the two days (*p*<0.01). Oxidative stress marker levels were maintained over all assayed samples with no difference at rest, indicating no effect of the exercise on the recovery periods on these values (Tables 2 and 3).

Percentage changes in MDA due to exercise revealed a highly significant difference of greater than twofold amounts of MDA for both days, irrespective of events held in day (increases were 114.7±48% after first day events and 108±30.13% after second day events, *p*<0.01). On the other hand, 8OHdG changes were very low, but significant, after both days of competition (9.5±7.5% and 18.4±14.4%, respectively).

## Discussion

The present study’s purpose was to study assays for plasma MDA and urine DNA oxidation products (8OHdG) in order to determine the effects of heptathlon T1 (without beverage) competition on the human body during two successive days. We conducted this study because it is known that MDA possesses free-radical peroxidation, and excessive stress can induce DNA damage in the form of oxidized nucleosides, strand breaks, or DNA crosslinking. Possible consequences of DNA damage are defective repair, apoptosis, and necrosis (Chevion et al., 2003). Another purpose was to investigate cell integrity after a course of strenuous efforts: four events on the first day, the 100m run, high jump, shot put, and 200m run, and another three on the second day, the long jump, javelin throw, and 800m run.

The significant increase in urine 8OHdG after exercise and its disappearance within 14h after exercise suggests that 8OHdG could possibly be an important tool for monitoring and quantification of cellular damage.

This study had three main results. The first is the twofold elevation of plasma MDA compared to only 9.5 or 18% elevation in urinary 8OHdG. Of course, highly elevated plasma MDA can be considered a result of the peroxidation of cell membrane lipoproteins and lipids, but this may be counteracted by enhanced re-synthesis without cell death. Also, increased adipose lipolysis for energy generation may add extra lipid peroxidation. Therefore, it makes sense that lipid peroxidatioon products are highly elevated. The relevance of antioxidant response versus peroxidation effect is geared toward 8OHdG rather than MDA.

It has been reported that a higher metabolic rate with increased oxygen consumption involves an increase in the urinary excretion of 8-OHdG. Inoue et al. (1993) reported that the ratio of urinary 8-OHdG to creatinine excretion increased in swimmers and runners after regular training. In addition, Alessio et al. (2000) also observed a significant increase in the ratio of urinary 8-OHdG to creatinine excretion 10h after a marathon race in runners. They suggested that antioxidant supplementation may provide better results.

Another result of this study is the decreased urinary 8-OHdG in second day samples when compared to baseline samples. This decline may be due to high anabolic activity during recovery. Sumida et al. (1997) suggested that the oxidative stress during a single bout of intensive exercise does not result in accumulation of oxidative DNA damage. Hartman’s data suggested that DNA effects detected with the comet assay in human leukocytes after exercise are secondary effects that do not originate from oxidized DNA bases and do not result in chromosome damage (Hartman et al., 1998).

The third significant outcome of the study is the high standard deviation values obtained with means after effort. This result indicates a wide individual variation in the athletes’ response to the exercise effort of the second day, although they are equivalent in their anthropometric measurements and training experience. Other causes may be the small number of participants involved in this study or the fact that the 800m run event is considered to be one of the most intensive exercises of the heptathlon.

## Conclusion

The conclusion of the present study is that urinary 8-OHdG could be considered as a novel biomarker in the quantification of cellular damage. The finding that the heptathlon can introduce higher cellular damage in the second day exercise set may be due to the 800m run event. Although urinary 8-OHdG levels are elevated in much lower concentrations compared to plasma MDA levels, antioxidant supplements may be beneficial for heptathlon athletes.

## Figures and Tables

**Table 1:** Anthropometric characteristics of the study participants (N=8)

Anthropometric character	Mean	SD	Skewness
Age (years)	22.9	4.26	−0.085
Body mass (kg)	58.5	4.45	−0.415
Body height (cm)	164.6	3.84	−1.263
Training experience (years)	8.7	2.69	0.861

**Table 2:** Markers of oxidative stress for heptathlon athletes following competition (Mean±SD)

	**Day 1**	**Day 2**
	Mean ± SD	
Plasma MDA Rest (μmol/l)	22.5 ± 2.33	24.37 ± 1.60
Plasma MDA effort (μmol/l)	47.5 ± 6.61	50.50 ± 5.63
Urine 8-OHdG Rest (nmol/l)	23.78 ± 1.95	22.96 ± 2.10
Urine 8-OHdG effort (nmol/l)	25.96 ± 1.33	27 ± 2.1

MDA=Malondialdehyde 8-OHdG=8-hydroxy deoxy guanosine

**Table 3:** Wilcoxon signed ranks for MDA and 8-OHdG with percent differences in investigated hepathlon athletes

	**%**	**Z**	**P**
MDA effort 1 - MDA Rest 1	114.7 ± 48	−2.52	0.01
MDA effort 2 - MDA Rest 2	108 ± 30.13	−2.52	0.01
MDA Rest 2 - MDA Rest 1	9.4 ± 13.89	−1.56	0.12
MDA effort 2 - MDA effort 1	8.3 ± 19.08	−1.18	0.24
8-OHdG effort 1 - 8-OHdG Rest 1	9.5 ± 7.51	−2.52	0.01
8-OHdG effort 2 - 8-OHdG Rest 2	18.4 ± 14.4	−2.38	0.01
8-OHdG Rest 2 - 8-OHdG Rest 1	−3.1 ± 9.47	−0.85	0.40
8-OHdG effort 2 - 8-OHdG effort 1	4.1 ± 7.36	−1.52	0.13

1=day 1 2=day 2 Z is significant at p≤0.05
